# “Maybe I’m not that approachable”: using simulation to elicit team leaders’ perceptions of their role in facilitating speaking up behaviors

**DOI:** 10.1186/s41077-022-00227-y

**Published:** 2022-09-24

**Authors:** Rachael Pack, Lauren Columbus, Trevor Hines Duncliffe, Harrison Banner, Priyanka Singh, Natashia Seemann, Taryn Taylor

**Affiliations:** 1 Centre for Education Research & Innovation, Schulich School of Medicine & Dentistry, Western University, London, Canada; 2 Department of Midwifery, London Health Sciences Centre, London, Canada; 3 Department of Obstetrics & Gynaecology, Schulich School of Medicine & Dentistry, Western University, London, Canada; 4 Schulich School of Medicine & Dentistry, Western University, London, Canada; 5 Department of Surgery, Schulich School of Medicine & Dentistry, Western University, London, Canada

**Keywords:** Simulation, Team communication, Leadership, Approachability

## Abstract

**Background:**

Simulation research that seeks to solve the problem of silence among interprofessional teams has focused almost exclusively on training subordinate team members to be more courageous and to speak up to team leaders using direct challenge scripts despite the great interpersonal cost. Consequently, the existing literature overemphasizes the responsibility of subordinate team members for speaking up and fails to consider the role and responsibilities of team leaders in sustaining silence. The purpose of this study is to identify and describe the subtle behaviors and actions of team leaders that both promote and discourage speaking up.

**Methods:**

This study used a simulation-primed qualitative inquiry approach. Obstetricians (OB) at one academic center participated in an interprofessional simulation as an embedded participant. Five challenge moments (CM) were scripted for the OB involving deliberate clinical judgment errors or professionalism infractions. Other participants were unaware of the OB embedded participant role. Thirteen iterations were completed with 39 participants. Twelve faculty members completed a subsequent semi-structured interview. Scenarios were videotaped; debriefs and interviews were audio-recorded and transcribed verbatim. Data were analyzed using an inductive thematic approach.

**Results:**

After participating in an interprofessional simulation, faculty participants reflected that being an approachable team leader requires more than simply avoiding disruptive behaviors. We found that approachability necessitates that team leaders actively create the conditions in which team members perceive that speaking up is welcomed, rather than an act of bravery. In practice, this conceptualization of approachability involves the tangible actions of signaling availability through presence, uncertainty through thinking aloud, and vulnerability through debriefing*.*

**Conclusions:**

By using faculty as embedded participants with scripted errors, our simulation design provided an ideal learning opportunity to prompt discussion of the subtle behaviors and actions of team leaders that both promote and discourage speaking up. Faculty participants gained a new appreciation that their actions create the conditions for speaking up to occur before critical incidents through their verbal and non-verbal communication.

**Supplementary Information:**

The online version contains supplementary material available at 10.1186/s41077-022-00227-y.

## Background

Safe teamwork demands a shared willingness to speak up [1–3]. However, interprofessional healthcare teams often struggle with fully embracing this premise [1–5]. In medicine, the dominance of a culture that values projecting confidence, certainty, and hiding vulnerability has created an “epidemic of silence” [6], p.30 due to fear of being wrong, disrespectful, or ostracized [1, 5]. Simulation research that seeks to solve the problem of silence among interprofessional teams has focused almost exclusively on training subordinate team members (i.e., residents and nurses) to be more courageous and to speak up to team leaders using direct challenge scripts [4, 5, 7–9] despite the great interpersonal cost [1, 6]. As a consequence of this focus, the existing literature overemphasizes the responsibility of subordinate team members for speaking up and fails to consider the role and responsibilities of team leaders in sustaining silence.

Communication is a cornerstone of patient safety [10]; the failure of effective communication among healthcare teams has been strongly linked to adverse events and medical errors [11]. These events are not infrequent; a recent study of surgical residents reported that 49% of residents observed a patient safety breach in the preceding month [12]. Despite the frequency of these events, research has consistently shown that residents and other members of interprofessional teams often remain silent rather than convey their concern to the team leader [4, 5, 9]. As high-status members of the hierarchy, team leaders play a significant role in shaping the culture of their team and the types of action and inaction expected of its members. Disconcertingly, previous research demonstrates that when team members do speak up, they are often ignored [3]. When team leaders fail to acknowledge the concerns of team members or “listen down,” both patient safety and team performance suffer [13].

Importantly, the effects of a leader’s behavior on the dynamics of their team are not always visible to the individual. Previous research has shown that subtle changes in the behavior, language, and demeanor of faculty can dramatically affect trainees’ willingness to speak up [9] and that team leaders are often unaware of the deleterious effects of their behavior [9, 13]. Additionally, the way that team leaders respond to challenges also may affect future speaking up behaviors. Existing research has demonstrated that nurses whose prior speaking up efforts were received favorably were more likely to speak up in the future [2]. Careful work is required to further identify and describe the subtle behaviors and actions of team leaders that both promote and discourage speaking up [9].

Previous work on interprofessional teams reveals that in deeply hierarchical environments, fear of retribution, fear of being wrong, and intimidation are key factors that prevent team members (e.g., residents and nurses) from challenging decisions that negatively affect patient safety [1, 2, 8, 14]. This is typical of settings that lack psychological safety, which Edmondson [15] defines as “shared belief that the work environment is safe for interpersonal risk taking” p.350. Yet we know that unacceptable behaviors that make interprofessional risk taking unsafe, including speaking up, are rampant in healthcare settings [13]. As Edmondson acknowledges simply, “exhorting people to speak up because it’s the right thing to do relies on an ethical argument but is not a strategy for ensuring good outcomes” [6] p.82. Yet, research and education interventions remain focused on encouraging these team members to speak up in spite of their fears and, in many cases, legitimate interpersonal risk [16]. These interventions have been largely unsuccessful in sustainably resolving issues around speaking up in the clinical workplace [17] as they fail to address the power differentials that are ingrained in the culture of healthcare teams [2, 5, 6, 18].

To effectively disrupt the culture of silence in medicine, we need to broaden our attention from focusing exclusively on subordinates’ role in speaking up to understand what behaviors team leaders can engage in to support a culture of effective and open team communication. This study uses simulation-primed inquiry in a multi-professional obstetrical scenario to elicit team leaders’ perspectives on their role in establishing and fostering an environment in which team members feel safe to speak up and advocate for patient care.

## Methods

We used a simulation-primed qualitative inquiry approach [19], which is well-suited to our socially situated research question, because it facilitates a candid exploration of sensitive or emotionally charged experiences. This approach uses simulation as an elicitation strategy, which primes participants to prompt reflection, rather than as an intervention.

### Setting, participants, and recruitment

We recruited participants via email and word of mouth from one tertiary (level III) academic hospital in Ontario, Canada, where obstetrician (OB) faculty members work alongside residents (postgraduate trainees), labor and delivery nurses, registered midwives, and family physicians. All practitioners in each of the aforementioned groups were eligible to participate; all those who chose to participate had worked together previously. Study participants are detailed in Table 1.

**Table 1:** Study participants

Simulation and debrief participants	Individual, semi-structured interview participants
13 faculty obstetricians	12 faculty obstetricians
2 family medicine obstetricians	2 family medicine obstetricians
11 obstetric residents	7 obstetric residents
5 midwives	2 midwives
8 nurses	7 nurses

In the study setting, midwives and family physicians manage their own clients and patients, respectively; they consult the OB on call if complications arise. Meanwhile, OBs concurrently manage both high- and low-risk laboring patients and obstetrical inpatients, along with residents and nurses, within a 24-h call model. Participation in the study was voluntary and participants received a $25.00 gift card honorarium for their participation. The study focus on the recruitment materials was kept deliberately generic, by stating that the study was focused on “interprofessional team dynamics and communication practices” to ensure the participants were unaware of the OB's embedded participant status, as outlined below. Ethics approval was provided by the Institutional Research Ethics Board (REB: 115446).

Our decision to situate this study in obstetrical multi-professional teams was pragmatic and purposive. It ensured that we could provide content expertise, to support learning objectives during the simulation scenario and debrief (led by TT, an obstetrician), while leveraging the lived experiences of TT, HB, and LC who work within these teams daily to inform the scenario design.

### Simulation scenario

TT and PS collaboratively designed the simulation scenario (Additional file 1) to reflect a realistic case involving an OB in a community-level hospital who was overwhelmed and distracted, as reflected by five scripted errors in judgment or professionalism, which we called “Challenge Moments” (CM). We deliberately chose a range of CM that were based on a composite of TT’s experience in training and practice. Other collaborators (HB and LC) and a current resident who was excluded from study participation all confirmed resonance with their lived experiences. Scripted errors are outlined in Table 2 and the instructions provided to embedded participants are included in Additional file 2. The OB participants were considered “embedded participants” because they were asked to commit scripted errors (challenge moments), but they did not receive any ongoing guidance or direction beyond that during the scenario. These embedded participants were expressly instructed to consider how they might engage in these scripted errors on a “bad night” rather than taking on a completely different persona and engaging in bullying or other overtly aggressive behavior.

**Table 2:** Scripted errors

Scenario element	Type of error	Scripted error
Responding to an urgent request for help	Unprofessionalism	Delayed response to call for help
Assessing fetal position	Clinical assessment	Incorrectly identify position
Supervising a forceps-assisted vaginal delivery	Procedural checklist error	Fail to ensure the proper safety steps are taken (e.g., consent, bladder drained, neonatal resuscitation team, and anesthesia available)
Recommend a contraindicated medication	Medication error	Request hemabate in active asthmatic patient
Abandon team in crisis	Lack of situational awareness	Leave the scenario prematurely to go attend to another patient

### Data collection and analysis

We conducted thirteen iterations of the simulation scenario in our simulation center with 39 participants from March to October 2021. Each group received a standardized pre-brief by TT, as most participants were simulation-naïve, including an orientation to the physical space and limitations of the mannequin, and to ensure confidentiality was maintained after the simulation. Scenarios were video and audio recorded. Aside from TT, the remaining co-investigators observed the scenarios and debriefs virtually, both synchronously and asynchronously, due to the COVID-19 pandemic. Following the scenario, all participants reconvened for a debrief led by TT, following the PEARLs debriefing model [20]; each session was recorded and transcribed verbatim. The deception regarding the OB embedded participant was disclosed at the beginning of the debrief; special attention was paid towards establishing and maintaining psychological safety throughout the debrief in light of the deception [21]. In the analysis phase of the debrief, all participant groups discussed the challenge moments. Because the analysis phase of the debrief was participant-driven, follow-up prompts from the debriefer (TT) were unscripted and intended to probe for clarification or elaboration only.

All participants were then invited to participate in a virtual individual, semi-structured follow-up interview at a mutually convenient time. Only participants who responded to the invitation and consented to participate were interviewed. Thirty participants (12 faculty members) completed a follow-up interview. These interviews were also recorded and transcribed verbatim. Where necessary, segments of the simulation video were shown to participants to elicit specific insights into their thoughts and decisions during the scenario. Debrief transcripts were similarly used to probe in more detail about relevant comments or reflections participants had made during the debrief.

In keeping with our qualitative approach, we proceeded with data collection and analysis iteratively. This enabled us to shift our sampling strategy and revise our follow-up interview guide to explore preliminary analytical insights as they developed. For example, after the first few scenarios involving a midwife, a resident, and an OB were completed, we expanded our recruitment to run the scenario with a family physician, a nurse, and an OB to shed light on the dynamics that occur when one team members shifts from being the most responsible provider (MRP) to a subordinate role with a distracted, overwhelmed OB. The simulation videos were used for elicitation purposes only and were not formally analyzed for the purposes of this study. Transcripts from the debriefs and follow-up interviews were analyzed using an inductive thematic approach [22]. RP and TT held regular analytical meetings to discuss developing insights, informed by the iterative thematic coding and alongside analytical memos and field notes written by other co-investigators while observing the scenarios and debriefs. Once a more conceptual coding structure was constructed, through constant comparative analysis, the entire research team met to review and further refine the relationships between key themes, resulting in the final analytical product.

### Reflexivity

Our interdisciplinary research team included two obstetricians (TT, HB), a midwife (LC), a sociologist (RP), a paramedic and PhD student (THD), and a medical student (PS). As a fellowship-trained simulation educator, qualitative researcher, and obstetrician, TT led the study design and implementation. The range of perspectives and lived experiences within the research team, each with varied insider/outsider status [23] relative to the study context, facilitated rich discussions about our assumptions and interpretation of the data.

## Results

We will elaborate on our findings outlined below with verbatim quotations from residents (R###), midwives (M###), nurses (N###), obstetricians (O###), and family physicians (F###) while indicating whether the quotation occurred during a debrief or follow-up interview.

### Approachability, redefined

The simulation experience prompted faculty to reflect on, and in some cases recalibrate, their sense of what it means to be an approachable team leader. We heard that many faculty previously viewed approachability as a fixed characteristic that was defined by the absence of overtly disruptive behaviors. At its most basic level, approachability meant behaving “in a civil, respectful manner” (OB010, interview) and responding to subordinates’ questions or errors in a non-punitive way. Another faculty member reflected that her team should “feel safe” asking her questions because she is not “a person who would try to make someone feel badly” (OB012, interview). In contrast, faculty defined unapproachability as a pattern of disruptive behavior and a demonstrated unwillingness to engage with the perspectives of others.

It’s the people who raise their voices, and who are kind of stubborn and unrelenting in their way of doing things. Or there’s people who you hear them talking about people behind their backs, criticizing the residents behind their backs, or even other consultants… I think, when you have that background of knowing how people talk about each other offline, sometimes gives you an idea of what those people’s responses are going to be. (OB009, interview)

While operating under this binary understanding of approachability, faculty participants anticipated that the errors and professionalism infractions they were scripted to enact during the simulation would be challenged by their team members; faculty were not instructed to engage in disruptive behaviors. However, interprofessional teams often went along with the unsafe and unprofessional behaviors set in motion by the faculty member.

Given this dissonance, faculty participants described their simulation experience as “eye-opening” (OB003, debrief) and “uncomfortable” (OB011, debrief). During the debrief, faculty grappled with the fact that their out-of-character behavior was seemingly accepted by the team: “nobody from the team questioned the ridiculousness that I was doing” (OB002, debrief). Faculty then sought to reconcile their simulation experience with their self-perceptions. This process is highly visible in the reflection of a faculty member, who revealed during their follow-up interview that they had been ruminating over the fact that “[the resident] didn’t challenge me on errors that I was making in judgement” during the simulation, and this sparked significant reflection about how they may be perceived by their team:

Maybe I’m not that approachable. Maybe they’re scared to disagree with me. I don’t think that’s the case at [this resident’s training] level, but I don’t know. Maybe I should reflect on it myself. (OB007, interview)

The experience also left some faculty wondering how these moments are playing out beyond the simulation context, since it was not inconceivable that they might make unintentional mistakes or errors in judgment in “an off-moment or an off-day” (OB 003, interview). As this participant went on to explain:

I would hope that someone would call me out on it and bring that to my attention and not just internalize it and rationalize it, that this is the way she normally works, so this is okay. Because we are human. We are all going to make errors. (OB003, interview)

As the above participant observed, the willingness of their team to trust their judgment even when it contradicted best practices was both “nice” and “scary” (OB003, interview). Many participants grappled with contradictory feelings about the willingness of their team to trust their judgment and the position of power they held at the top of the team hierarchy:

[The simulation is] a good example to me of how much power I wield, and how easy it is to railroad people into doing things that they’re not comfortable with, just because of the power dynamic. ...it’s kind of humbling to think I still have that much pull over what people do...So, if I’m not making the best, most educated, appropriate choices, that it has real downstream repercussions (OB009, debrief).

While most faculty participants thought of themselves as approachable, their experiences during the simulation and debrief challenged their sense of what it means to be approachable. It prompted faculty to confront the possibility that the absence of uncivilized behaviors, such as ridiculing team members or responding with disdain, may not be sufficient to create the conditions for speaking up to occur. Subordinate team members, on the other hand, were keenly aware that faculty perceptions of approachability may not be shared by the team and appreciated that the simulation experience prompted faculty to confront misperceptions.

[This simulation is] important for the consultants to see because I think they expect us to just feel comfortable speaking up about these things and that’s not the case (R004, interview)

Post-simulation interprofessional debriefs and subsequent individual interviews elicited reflections from all participants about notions of approachability and how it can be fostered in the clinical workplace. Through our analysis, we identified three overlapping building blocks of approachability: availability, uncertainty, and vulnerability. In practice, these building blocks were signaled through presence, thinking aloud, and debriefing, respectively.

### Availability and presence

Non-faculty participants spoke at length about how faculty members’ availability or unavailability influenced their perceptions of approachability. Availability, in this context, refers to the degree to which a faculty member was believed to be willing and able to engage as part of the team. Availability was communicated by physical presence in clinical and shared team spaces and a willingness to communicate about patient care. Despite the simplicity of physical presence on the labor and delivery unit, participants spoke about how this dimension of approachability was not so straightforward:

Physical presence is huge, hugely variable between staff…If you know that somebody is around…you know that that person wants to be involved and wants to hear…Then there’s some staff that are - you don’t see them. I was leaving the hospital one day, and I saw [an OB] in his car driving back into the parking garage. He had been on call for the birthing centre and was not physically in house... That is a person who I would not feel comfortable reaching out to, because he was literally not even in the hospital. (R009, interview).

The physical presence of faculty members particularly in common spaces (e.g., team rooms or lounges) was identified as another important modality of presence that helped to facilitate relationships among team members and, in turn, foster approachability.

There are staff that just kind of disappear and they’re only contact is, like, by phone, which in some ways makes them less approachable, because you don’t, like, bump into them and you don’t do small talk. When they’re kind of around in the team room ...it makes them feel like they’re kind of, we’re all part of a team. So, I think that makes them also a bit more approachable. (R012, interview)

Presence empowered team members to ask questions and expand their knowledge. As one nurse lamented, the presence of faculty members at the nursing station was previously something she “loved” since “the consultants would sit there and teach all the residents’ things. They would teach the nurses things. That doesn’t happen anymore” (N012, interview).

Team members also described how presence dismantled barriers to asking for help. One family medicine obstetrician recalled an experience with a difficult delivery when a faculty member’s physical presence and offer of assistance provided a lifeline:

I was watching an ugly heart rate and I had an OB poke their head in the room and just say, is everything okay, do you need me?... [My response was] if the baby is not out by the next one or two contractions, yeah, I would like you to help out. (FM008)

Faculty members demonstrated some awareness that their presence affected their perceived approachability and that being physically present “invites more of a dialogue” (OB007, interview) among the team. Despite this, faculty acknowledged that physical presence was not always feasible. As a result, some participants described strategies they developed to communicate their availability to the team in instances when they could not be present:

I hear this from the residents sometimes. They’re like well I wasn’t sure if I should call you or, I didn’t want to bother you or, I didn’t want to wake you… So, at the beginning of a call shift …Please call me any time, if you’re not sure about something or if you’re wondering about something, like don’t worry about waking me up. (OB011, interview)

Thus, as our participants described, approachability meant more than just showing up for deliveries or responding to pages; rather, it was an active process of maintaining visibility *between* those crucial moments and proactively establishing expectations when physical presence was not possible.

### Uncertainty and thinking aloud

The willingness of faculty to acknowledge clinical ambiguity or the “grey” in medicine was identified as a second building block of approachability. A willingness to acknowledge the ambiguity of medicine was described by one resident as “the most welcoming trait that a staff can have,” since “the staff that pretend that they know everything and there is no grey and there is black and white...that doesn’t open up any doors for questioning or having a discussion” (R003, interview).

This dimension of approachability was manifested through thinking aloud and sharing one’s mental model. One faculty member described how a memorable experience from their own training highlighted the importance of acknowledging practice variation.

There’s a very basic step of taking down the round ligament at a hysterectomy, [and the faculty member said] ‘if there are seven different ways of doing it, is there really one better way?’ Then it gets you thinking that there are a lot of things that you do, based on preference, superstition, comfort. (OB005, interview).

Recognizing practice variation, this faculty member described how they actively work to de-mystify their own preferences by sharing their rationale for why they prefer one technique over another:

I will just tell them, listen, I don’t think that there’s a right answer, but here’s the reasons why I’m thinking we should do this…Instead of doing all the thinking in my head, and just telling them the decision. (OB005, interview)

In striving to render their decision-making process transparent to their team, this faculty member acknowledged uncertainty in practice and created space for team members to ask questions. Participants recognized that sharing one’s thought process, particularly in ambiguous clinical situations, was a powerful tool to prevent disagreement and mistrust among the team. A nurse recounted a situation when they were unsure about the treatment plan put forward by an OB and reflected that the OB’s willingness to share their rationale resolved her apprehensions and restored confidence:

I can respect somebody saying, I do hear what you’re saying, but these are the reasons why I’m still making the decision that I’m making (N008, interview).

However, this participant lamented that faculty members often do not explicitly share their thought processes with the nursing staff.

To be honest, those rationales are probably put more towards the patient, so then I’m just overhearing them. Rather than put to me as the nurse. … [There have been only a few experiences] that I’ve had them actually tell me the rationale of why they’re doing what they’re doing. (N008, interview)

The absence of transparent decision-making was identified by participants as a key feature of unapproachability and a factor that contributed to poor team relationships. However, in instances where challenges were raised by team members, sharing one’s thought process was an effective strategy to resolve conflict among the team. One faculty member described how thinking out loud after a challenge from a subordinate gave her an opportunity to reflect on her rationale and affirm her judgment.

I think it’s important, when we are challenged, especially with those hierarchy roles, to be able to take a step back and say, okay, am I uncomfortable being challenged or is it making me uncomfortable because this is already a difficult scenario and it’s making me reflect on my decision-making? (OB003, interview)

While thinking aloud and expressing uncertainty were highly valued by team members, and learners in particular, some faculty participants highlighted the riskiness of doing so in their professional culture.

The culture of medicine is such that we’re supposed to be perfect, and of course, that isn’t true… I really struggled when I was a learner that it always seemed like the attending knew what to do in the surgery. They always seemed to know what the next step was. And it was retrospective reflection for me that I was like, that couldn’t have always been true, they just didn’t voice it. And so, one of my efforts, especially as a young staff, was to say, ‘I don’t know, truly.’ (OB004, interview)

Voicing their uncertainty and sharing their thought processes were identified by participants as tangible actions that faculty could engage in to communicate their approachability and willingness to fulsomely engage with the perspectives of their team members.

### Vulnerability and debriefing

Being vulnerable enough to admit an error or misstep by debriefing after unexpected outcomes was identified as another critical building block of approachability. Though this required explicit vulnerability on the part of the faculty leader, it was seen as an important strategy to maintain trust and effective communication among the team. The absence of debriefing or insufficient opportunities for team members, and learners in particular, to ask questions was identified as a significant contributor to poor team dynamics that extended well beyond the team members involved in a particular case.

[Residents] are supposed to learn from every single case. And if you can’t ask questions, or question anybody on why [something was done], how are you ever going to learn from it? If a resident felt that they couldn’t ask questions and they’re upset about how one thing goes it bleeds into everything else that they do. And then, all of a sudden, the problem is not between two people, the problem is between the 200 people. (N012, interview)

Team members particularly valued debriefing after critical clinical events and saw it as a tool to prevent conflict and maintain trust among the team, especially in cases where a team member disagreed about the course of treatment.

Debriefs are an opportunity to view the situation from all angles and to have an opportunity to actually walk through what each individual was thinking at the time. Because I often feel that you might be thinking one thing, but the staff is thinking a completely different thing. (R012, interview)

Team members appreciated when staff genuinely invited team members’ perspectives after a difficult case or unexpected outcome, because it dismantled narratives of the infallible (and thus unapproachable) team leader. One midwife reflected that anytime “there is a safe environment to be asking questions and providing feedback in both directions that, I think, really significantly changes the dynamics of how we talk to each other” (M010, interview).

On the other hand, team members were cautious of faculty members that presented themselves and their judgment as unimpeachable. Raising concerns or questions surrounding patient management with such a faculty member exposed team members to significant interpersonal risk:

They may be frustrated with the situation, but the way that they respond [to your questioning] makes it seem like they’re frustrated with you for expressing it. That definitely discourages you from reaching out to those people unless you feel you really have to, which I think is unsafe. (R010, interview)

In contrast, faculty members that embraced vulnerability were deliberate about drawing attention to decisions that exposed patients to risk, even in situations where the outcome was good.

I hate that feeling of, wow, I can’t believe I got away with that…That’s when a debrief with the residents, to talk about these processes, and why this was done in the moment, and why I might not do that again, is really important. (OB009, interview)

Faculty acknowledged that the pace of clinical work did not always allow them to think aloud with the team. In instances where decisions were made without transparency, some faculty recognized the importance of debriefing to repair any damage retrospectively. For example, a faculty member described a high-pressure situation where they decided to move the patient to the operating room without discussing their rationale with the team and reflected that their lack of transparency had “created an uncomfortable situation” for the team. To resolve this, they closed the loop after the event:

I’ll go back and say look, I’m sorry for that. This is what I was thinking. This is what I was worried about, and that’s why I did what I did. (OB007, interview)

The necessity of repair after instances where faculty members could not enact the modalities of approachability illustrates that approachability is not a static characteristic — it is in a state of constant becoming, produced through one’s actions and behaviors. It can be disrupted by difficult moments and reconstituted through reparative actions that employ the modalities of presence, debriefing, and thinking aloud. As one faculty member remarked, it is the “consistency in behavior across different realms or domains” (OB009, interview) that matters and gives team members confidence that you are a safe person to speak up to.

## Discussion

Participation in an interprofessional simulation designed with multiple scripted challenge moments elicited critical insights from faculty about what it means to be approachable and facilitate speaking up. Many participants described a realization that being an approachable team leader requires more than simply avoiding disruptive behaviors. We found that approachability necessitates that team leaders actively create the conditions in which team members perceive that speaking up is welcomed, rather than an act of bravery. In practice, this conceptualization of approachability involves the tangible actions of signaling availability through presence, uncertainty through thinking aloud, and vulnerability through debriefing. Furthermore, our data suggests that approachability is a dynamic state that is constantly reconstructed through action and more fragile than our faculty had originally assumed.

Approachability is an intangible concept in health professions education. As Deyo-Svendsen et al. [24] highlight, there exists no universally accepted, or empirically derived, definition of approachability within healthcare teams. As a starting point, their group defined approachability as the “words and actions that promote trust and reduce or eliminate fear of interaction” [24], p.e64. Our study provides empirical evidence that affirms this definition and illuminates the dynamic and fragile nature of the construct. Deyo-Svendsen and colleagues hinted at the fragile nature of approachability when they wondered about “the ‘staying power’ of past experiences when trying to measure and improve current perceptions of approachability” [24], p.e68. Our work suggests that previous experiences in which team leader approachability is compromised not only have staying power, but can also influence other team members’ perceptions of the leader’s approachability, regardless of whether the other team members were present or involved. Thus, based on our findings, we would advocate for an expanded definition of approachability that reflects the tenuous nature of approachability and illustrates the importance of reparative action (i.e., de-briefing) when approachability has been breached.

In reflecting on their beliefs about approachability prior to the simulation experience, our faculty participants were united in their belief that unacceptable behavior, or “negative behavior that violates the norms of mutual respect” [13], p1 is harmful to team communication. Unfortunately, mounting data expose the concerning prevalence of such unacceptable behaviors within healthcare teams [13]. There is robust evidence that unacceptable behaviors affect individual performance and productivity while also negatively impacting patient outcomes [13]. Our findings complicate our current understanding of unacceptable behavior as it relates to approachability. Approachability is not just about maintaining the norms of mutual respect, but it is also about engaging in behaviors that demonstrate that the perspectives and offerings of the team are valued. It is possible that failing to engage in behaviors that constitute approachability may have some of the same negative implications as engaging in unacceptable behavior, particularly as it relates to silence in the face of a patient safety threat. Depending on the pre-existing relationship and power differentials, even subtle cues from the team leader may preclude approachability [9]. If team leaders assume that other team members will speak up to them as long as they are not engaging in overtly unacceptable behavior, as our faculty members believed, then the silence within interprofessional teams is likely to persist.

Thus, our findings support the notion that approachability matters as it relates to facilitating speaking up behavior. Failure to speak up is not an issue of unmotivated team members. Individuals have difficulties speaking up even in situations where there is likely or certainly to be serious patient harm [4, 8, 25]. Team members who remain silent (or are silenced), despite anticipating patient-related harm often experience moral distress and guilt [4, 25, 26]. Many scholars have instead framed silence as a cognitive issue that can be overcome in a simulated setting if team members are taught the correct script to use [5, 8, 25]. Researchers have also indicated cognitive barriers to speaking up, which include: lack of self-esteem [27], deference to authority [28], and self-efficacy [7]. Thus, the focus, and in some cases the blame, is placed squarely on the shoulders of team members who are called to be more confident, to challenge authority, and to just believe that their courageous actions will have an impact. The longevity of these interventions, and the likelihood that they will translate to increased speaking up behavior in real life, remains uncertain [29]. While we fully support training efforts that aim to empower team members to speak up, or to find the most effective words during a challenge moment, our research strongly suggests that we need to recalibrate our focus and see speaking up as a responsibility shared among the entire team.

Given that team leader approachability also enables speaking up behaviors, targeted faculty development is long overdue. We must ensure that team leaders are supported in finding ways to actively establish approachability and sharpen their ability to listen down. For example, in our study, team leaders who were present and pre-emptively invited team members to ask questions as they arose were identified as approachable. Similarly, team members appreciated leaders who routinely acknowledge practice variation and shared their mental model aloud, particularly when deviating from standard or conventional practices. Many participants recognized that acute situations did not always allow for a thorough team discussion in the moment; in such cases, we heard that debriefing, when conducted with humility and candor, re-established perceptions of approachability. Collectively, these strategies are relatively easy solutions to an otherwise complex problem, a problem that Edmondson calls the “epidemic of silence” [6], p.30. While they demand some time, energy, and most importantly critical insight from team leaders, they offer the potential for profound impact.

### Limitations

This study took place in a single institution during a global pandemic, which limited the number of participants per simulation session. We tried to overcome possible threats to realism, and enhance buy-in, by situating the scenario in a community-based practice setting where three providers at the patient bedside would be common, even in an acute situation. Optimizing realism was a priority because the simulation was used as an elicitation prompt in our study design and we wanted the experience to feel as authentic as possible to spark candid reflections on the nature of silence and speaking up both inside and outside of the simulated environment. We utilized moments from the simulation to elicit participants’ reflections on their thought process and focused our analysis on the debrief and interviews that followed the simulation. Our methodology does not allow us to make any claims about how closely our participants’ actions in the simulation exercise resemble their real-life practice. However, participants’ reflections throughout the debrief and follow-up interview process suggest that their responses during the simulation resonated with their prior experiences. Follow-up interview participation was voluntary and thus it is possible that those who chose not to complete a follow-up interview may have had different perspectives to offer. Based on the chosen methodology, we are unable to generalize beyond the study context, though we intend to pursue future research in other contexts to explore the transferability of our findings. We invite readers to decide for themselves whether our findings resonate with their lived experience in their contexts.

## Conclusions

By using faculty as embedded participants with scripted errors, our simulation design prompted a candid exploration of the subtle behaviors and actions of team leaders that both promote and discourage speaking up. Faculty participants gained a new appreciation that their actions create the conditions for speaking up to occur before critical incidents through their verbal and non-verbal communication.

## Supplementary Information

**Additional file 1.** Hierarchy simulation.

**Additional file 2.** Instructions for partial confederates.

## Data Availability

The qualitative dataset generated during and analyzed during the current study are not publicly available because they contain identifiable information about our participants. De-identified data and study materials may be made available from the corresponding author on reasonable request.

## Abbreviations

CM: Challenge moments
OB: Obstetrician
MRP: Most responsible provider

## References

[1] Landgren R, Alawadi Z, Douma C, Thomas EJ, Etchegaray J (2016). Barriers of pediatric residents to speaking up about patient safety. Hosp Pediatr.

[2] Maxfield D, Grenny J, Lavandero R, Groah L. The silent treatment: why safety tools and checklists aren't enough to save lives. American Association of Critical-Care Nurses (AACN), the Association of periOperative Registered Nurses (AORN), VitalSmarts; 2011. Available from: http://www.silenttreatmentstudy.com/.

[3] Okuyama A, Wagner C, Bijnen B (2014). Speaking up for patient safety by hospital-based health care professionals: a literature review. BMC Health Serv Res.

[4] Friedman Z, Perelman V, McLuckie D, Andrews M, Noble LM, Malavade A, Bould MD (2017). Challenging authority during an emergency—the effect of a teaching intervention. Crit Care Med.

[5] Bould MD, Sutherland S, Sydor DT, Naik V, Friedman Z (2015). Residents’ reluctance to challenge negative hierarchy in the operating room: a qualitative study. Can J Anesth.

[6] Edmondson AC (2018). The fearless organization: creating psychological safety in the workplace for learning, innovation, and growth.

[7] Guris RJ, Duarte SS, Miller CR, Schiavi A, Toy S (2019). Training novice anaesthesiology trainees to speak up for patient safety. Br J Anaesth.

[8] Pian-Smith MC, Simon R, Minehart RD, Podraza M, Rudolph J, Walzer T, Raemer D (2009). Teaching residents the two-challenge rule: a simulation-based approach to improve education and patient safety. Simul Healthc.

[9] Salazar MJ, Minkoff H, Bayya J, Gillett B, Onoriode H, Weedon J, Altshuler L, Fisher N (2014). Influence of surgeon behavior on trainee willingness to speak up: a randomized controlled trial. J Am Coll Surg.

[10] Kohn LT, Corrigan JM, Donaldson MS (2000). To err is human: building a safer health system.

[11] Lingard L, Espin S, Whyte S (2004). Communication failures in the operating room: an observational classification of recurrent types and effects. Qual Saf Health Care.

[12] Martinez W, Lehmann LS, Thomas EJ (2017). Speaking up about traditional and professionalism-related patient safety threats: a national survey of interns and residents. BMJ Qual Saf.

[13] Guo L, Ryan B, Leditschke IA, Haines KJ, Cook K, Eriksson L, et al. Impact of unacceptable behavior between healthcare workers on clinical performance and patient outcomes: a systematic review. BMJ Qual Saf. 2022. 10.1136/bmjqs-2021-013955. 10.1136/bmjqs-2021-013955 35046101

[14] Belyansky I, Martin TR, Prabhu AS (2011). Poor resident attending intraoperative communication may compromise patient safety. J Surg Res.

[15] Edmondson A (1999). Psychological safety and learning behavior in work teams. Adm Sci Q.

[16] Edmondson AC (2003). Speaking up in the operating room: how team leaders promote learning in interdisciplinary action teams. J Manag Stud.

[17] Pattni N, Arzola C, Malavade A, Varmani S, Krimus L, Friedman Z (2019). Challenging authority and speaking up in the operating room environment: a narrative synthesis. Br J Anaesth.

[18] Janss R, Rispens S, Segers M, Jehn KA (2012). What is happening under the surface? Power, conflict and the performance of medical teams. Med Educ.

[19] Wong AH, Tiyyagura GK, Dodington JM, Hawkins B, Hersey D, Auerbach MA (2017). Facilitating tough conversations: using an innovative simulation-primed qualitative inquiry in pediatric research. Acad Pediatr.

[20] Eppich W, Cheng A (2015). Promoting Excellence and Reflective Learning in Simulation (PEARLS): development and rationale for a blended approach to health care simulation debriefing. Simul Healthc.

[21] Kolbe M, Eppich W, Rudolph J, Meguerdichian M, Catena H, Cripps A (2020). Managing psychological safety in debriefings: a dynamic balancing act. BMJ Simul Technol Enhanc Learn.

[22] Braun V, Clarke V (2006). Using thematic analysis in psychology. Qual Res Psychol.

[23] Dwyer SC, Buckle JL (2009). The space between: on being an insider-outsider in qualitative research. Int J Qual Methods.

[24] Deyo-Svendsen ME, Palmer KB, Albright JK, Phillips MR, Schilling KA, Svendsen ME (2019). Provider approachability: an all-staff survey approach to creating a culture of safety. J Patient Saf.

[25] Sydor DT, Bould MD, Naik VN (2013). Challenging authority during a life-threatening crisis: the effect of operating theatre hierarchy. Br J Anaesth.

[26] Dzeng E, Wachter RM (2019). Ethics in conflict: moral distress as a root cause of burnout. J Gen Intern Med.

[27] Etchegaray JM, Ottosen MJ, Dancsak T, Thomas EJ (2020). Barriers to speaking up about patient safety concerns. J Patient Saf.

[28] Cosby KS, Croskerry P (2004). Profiles in patient safety: authority gradients in medical error. Acad Emerg Med.

[29] Kim S, Appelbaum NP, Baker N, Bajwa NM, Chu F, Pal JD, Cochran NE, Bochatay N (2020). Patient safety over power hierarchy: a scoping review of healthcare professionals’ Speaking-up skills training. J Healthc Qual.

