# Supplementary material for: “Maybe I’m not that approachable”: using simulation to elicit team leaders’ perceptions of their role in facilitating speaking up behaviors
Source: Adv Simul (Lond). 2022 Sep 24;7:31. doi: 10.1186/s41077-022-00227-y (PMC9509643; doi:10.1186/s41077-022-00227-y)
Supplement: Supplementary file 2 — Additional file 2. Instructions for partial confederates. [file 41077_2022_227_MOESM2_ESM.pdf]

Instructions for Partial Confederates

You are the obstetrician on call in a busy community (level II) hospital. You are aware that there is patient on the board who has been slow to progress. You've had a stressful, busy night so far and you're exhausted and a bit irritated. When you are called to assess the patient, as part of the scenario, we would like you to do the following:

1. **When called by OB resident, your initial response will be that you will come when you're free, purposely ambiguous.** (Backstory here is that you just finished dealing with a challenging interaction in triage)

2. **Incorrectly assess the baby's position to be OA (it will be OP) (**it's a difficult check due to molding/caput.)

3. **Decide to perform a forceps assisted vaginal delivery without conducting patient consent first and without emptying the bladder or calling for peds resuscitation team** (you can initially act as though the urgency of the situation precludes these steps)

4. **Only provide assistance to the OB resident with the forceps delivery if directly requested**

5. **There will be a PPH following delivery (will occur in part two of the sim in a separate room). Request hemabate** (you don't know that the patient has a history of asthma)

6. **After the hemabate is administered, remove yourself from the room as the hemorrhage is ongoing and intensifying, stating to the team "I'm going to check on room 220, looks like you've got this under control"** (there's a patient who's been fully and pushing for 3 hours)

Most importantly, the other participants <u>cannot</u> know that you've been given these directions for us. In simulation, this is called being a partial confederate. Outside of these specific elements, we want you to respond and act however you normally would. We've provided (what we think are) realistic backstory info to explain why someone might initially, reasonably choose to do/say these things in a real-life situation. If the other team members question or clarify or challenge you, please feel free to respond as you normally would.
